# Impact Resistance of Aluminum Foam Composites with Filler and Coating Materials

**DOI:** 10.3390/polym16233286

**Published:** 2024-11-26

**Authors:** Yue Wu, Yulin Guo, Songwen Yi, Zhuwen Lv, Zhiqiang Fan

**Affiliations:** 1 School of Mechanical and Electrical Engineering, North University of China, Taiyuan 030051, China; 2 China Academy of Ordnance Science Ningbo Branch, Ningbo 315103, China; 3 State Key Laboratory of Dynamic Measurement Technology, North University of China, Taiyuan 030051, China

**Keywords:** impact resistance, composites, fracture, aluminum foam, coating

## Abstract

The main objective of this study is to analyze the impact resistance of aluminum foam composites containing fillers and coatings and to investigate the effect of different thickness ratios of the composites on this capability. We prepared composites using aluminum foam and polyurea and performed impact tests and numerical simulations. A comparison of the results shows that the Abaqus simulation results are in general agreement with the test results. The results show that the polyurea filler material and polyurea coating can significantly improve the impact resistance of the aluminum foam, and the best impact resistance of the aluminum foam composite with polyurea coating on the back. An extended study of the composites was carried out using a numerical model validated by the test results. For the energy absorption effect of the aluminum foam composites in the impact resistance process, there is an optimum value for the thickness ratio of the aluminum foam/polyurea composite, which is 3:1. The remaining kinetic energy of cylindrical fragments in the 3-1-1-2 composite material decreased by 13.26%, in the 4-1-1-2 composite material decreased by 11.91%, in the 2-1-1-2 composite material decreased by 11.78%, and in the 1-1-1-2 composite material increased by 2.7% when compared to the remaining kinetic energy of cylindrical fragments in the control group. The energy absorption efficiency of the aluminum foam composite increases as the residual kinetic energy of the cylindrical fragments decreases. The 3-1-1-2 composite can significantly improve the energy absorption effect, which can be used as a reference for the design of impact-resistant composites in the future.

## 1. Introduction

In today’s highly evolving engineering field, material performance optimization and innovation have always been key factors in driving technological progress [1,2,3]. Among them, the impact resistance of materials and their ability to absorb impact energy are crucial in numerous application scenarios, such as automotive crash safety, aerospace structural protection, and military protective equipment [4,5,6]. Metallic materials are widely utilized in the field of impact resistance and their preparation processes are well established. However, metallic materials are usually dense, which increases the weight of the component [7,8,9]. Excessive weight may increase the impact force and energy transfer, resulting in the weakening of its impact resistance [10]. Therefore, structural improvement of metallic materials has become a major challenge in manufacturing lightweight materials with excellent impact resistance [11].

Foam structures are discontinuous structures with pores, and their porous structure gives them good impact resistance [12,13,14]. Combining aluminum metal and foam structure can make aluminum foam, which is a lightweight material with very low density [15]. Under impact loading, aluminum foam can effectively absorb the impact energy and reduce the impact of the impactor on the surrounding environment and other components [16]. At present, the research on aluminum foam in the field of impact resistance is still relatively small, and the research on the impact resistance of honeycomb porous structures has achieved good results [17]. For example, someone has studied the effects of the velocity of the intruder and the shape of the slug on the impact resistance and intrusion energy of aluminum honeycomb core sandwich panels using the intrusion test [18]. Someone once carried out a vertical impact test on the material of the honeycomb sandwich structure and obtained a numerical solution for the ballistic limiting velocity of the round-ended projectile intruding into the honeycomb sandwich structure [19]. Someone has conducted a numerical simulation study on the impact resistance of sandwich structures with metal fiber materials as the core layer under bullet impact and found that the energy absorbed by the sandwich structure under high-speed impact first decreases and then increases with the increase of the impact velocity of the projectile [20]. Although aluminum foam is advantageous in terms of lightweight properties, it has low shear strength and provides weak protection against high-impact pressures [21,22,23]. Therefore, materials with excellent adhesion, abrasion resistance, and impact resistance are coated on the surface of aluminum foam to improve the impact resistance of the material [24].

Polyurea is a high-performance polymer generated by the reaction of isocyanate and polyol, which is characterized by fast curing, high abrasion resistance, high impact resistance, and excellent chemical resistance [25,26,27]. At present, the research on the combined use of polyurea and porous metal is still relatively small, and the composite materials coated with polyurea on dense metal have been verified to have good impact resistance [28,29,30]. For example, D. Mohotti [31] studied the impact resistance of polyurea-coated aluminum plate structures to low-speed bullets. The coating position was limited to the face of the bullet, with coating thicknesses of 6 mm and 12 mm, and aluminum plate thicknesses of 3 mm and 5 mm. A flat-head cylindrical bullet with a diameter of 37 mm and a weight of 5 kg hit the target plate at a speed of 5–15 m/s. The experimental results measured the anti-bullet performance of the aluminum plate by the degree of deformation on the back of the plate, and it was concluded that the face of the bullet coating could significantly reduce the deformation of the aluminum plate, and the thicker the coating, the better the effect. Y. X. Jiang [32] used a drop hammer experiment to load the polyurea-coated steel plate structure, and the loading effect was consistent with that of a flat-headed cylindrical projectile. The results showed that the polyurea coating could effectively improve the energy absorption capacity of the steel plate. When the coating thickness remained the same, the improvement effect was ranked from high to low as the facing surface coating, double-sided coating, and back surface coating.

The combined use of aluminum foam and polyurea has been initially demonstrated in current work. For example, Jiang Yuexin [32] studied the enhancement mechanism of combining polyurea coatings and steel plates and found that polyurea coatings can significantly affect the impact resistance of metallic materials. Her research results show that, when coated with the same thickness of polyurea layer, the ultimate energy absorption capacity of composite panels is in decreasing order: top-side coated panels, double-sided coated panels, and bottom-side coated panels. In order to quantify the energy absorption efficiency of the composite panels, a parameter E-d was proposed, and it was found that the composite panels coated with 4 mm polyurea on the upper side had the largest E-d value among all the panels. Guo Hui [33] has verified the enhancement mechanism of polyurea coating on aluminum foam to some extent. His research results showed that the presence of the polyurea coating resulted in uniform stress transfer to the walls of the aluminum foam holes, which effectively prevented damage caused by the splashing of aluminum foam fragments under high-speed impact loads. Coating polyurea on the stressed face of the aluminum foam specimen can effectively increase the yield stress of the composite specimen under dynamic loading so that the specimen still maintains good integrity after failure. However, none of these existing research efforts have investigated the enhancement of aluminum foam when polyurea fillers are combined with polyurea coatings. Therefore, the polyurea is filled into the holes of the aluminum foam to form an aluminum foam composite material containing filler, and then the polyurea is sprayed on the surface of the aluminum foam composite material to form a tough polyurea coating [34,35,36]. Aluminum foam composites with fillers and coatings are very new applications in research and are improving the impact resistance of composites [37,38,39].

In this paper, two types of aluminum foam composites with fillers and coatings are prepared from polyurea and aluminum foam, and numerical modeling and impact tests are carried out on single-layer aluminum foam materials and aluminum foam composites. The impact resistance of the aluminum foam composites with fillers and coatings under the impact of cylindrical fragments is investigated by comparing the numerical simulation results with the test results in Abaqus. The numerical model validated by the test results is used to extend the study of aluminum foam composites. The extended study is on the effect of different thickness ratios of aluminum foam/polyurea composite on impact resistance, aiming to provide new ideas for the study of the impact resistance of composites.

## 2. Impact Tests

### 2.1. Test Preparations

**Figure 1:**
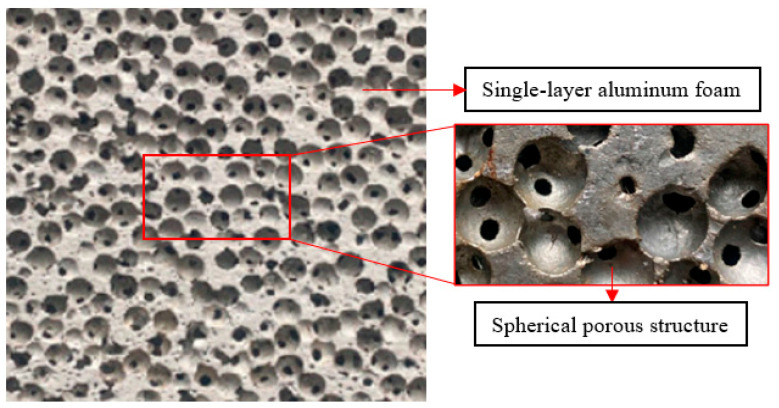
Single-layer aluminum foam and spherical porous structure.

The single-layer aluminum foam panels utilized in the test had spherical porous features that can effectively absorb impact energy [40]. The spherical porous structure is made of pure aluminum with 99.7% aluminum content, and the outstanding moldability of pure aluminum allows the aluminum foam to be easily created using the space support method. First, a space holder is fabricated using a metal mesh that should match the shape and size of the aluminum foam material to be prepared. Next, the space holder with the pure aluminum material is placed in a heating furnace, where it is heated and melted. During the heating treatment, an inert gas is introduced into the heating furnace to cause the pure aluminum to undergo a foaming reaction at high temperatures to form the foam aluminum material. Finally, the prepared aluminum foam material is removed from the heating furnace and cooled. The cooled aluminum foam material is removed from the space holder and the space holder is removed. As illustrated in Figure 1, a single-layer aluminum foam sheet measuring 200 × 200 × 30 mm is made up of spherical cells with an average diameter of 6 mm and holes 1–2 mm in size between the cells. Because the molds used to manufacture the test material had a porosity of 30–39%, the single-layer aluminum foam sheet was hypothetically prepared with a porosity of 61–70%. In this test, the apparent density of the single-layer aluminum foam sheet was roughly 0.9 g/cm^3^, implying that its real porosity was around 66%. The porosity comparison results reveal that the measured values for the single-layer aluminum foam panels before filling and coating are generally consistent with the theoretical results.

**Figure 2:**
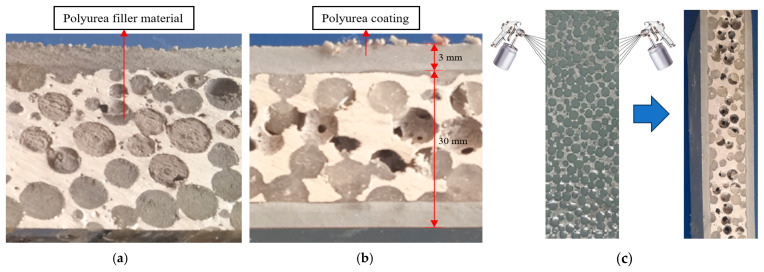
Aluminum foam composite with filler and coating. (**a**) Single-sided polyurea coating; (**b**) double-sided polyurea coating; (**c**) preparation of composite materials.

Because polyurea has good impact resistance, the aluminum foam composite was created by injecting polyurea into the holes of single-layer aluminum foam and adding a 3 mm polyurea coating to its surface. As demonstrated in Table 1, APC-40 type polyurea had much higher mechanical qualities than the other two, hence it was selected as the filler material and coating. As shown in Figure 2, two interpenetrating phase composite (IPC) structures of aluminum foam composites, single-side polyurea coating and double-side polyurea coating, with dimensions of 200 × 200 × 33 mm, were prepared using both infiltration and spraying. The interpenetrating phase composite (IPC) structure is generated by pressing the polyurea elastomer into the spherical cells with a piston. The composite was removed from the mold before the polyurea in the spherical cells had fully cemented, and a 3 mm thick polyurea coating was created by spraying APC-40 type polyurea on its surface many times. The existence of a high number of minute holes in the polyurea coating was caused by the small amount of air that was inevitably mixed in during the repeated sprays. Overall, the spherical porous aluminum skeleton, filler material, and coating demonstrated a strong connection.

**Figure 3:**
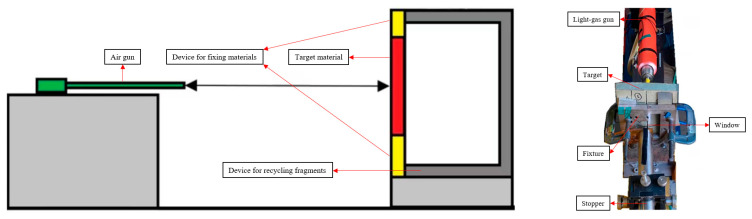
Test setup.

**Figure 4:**
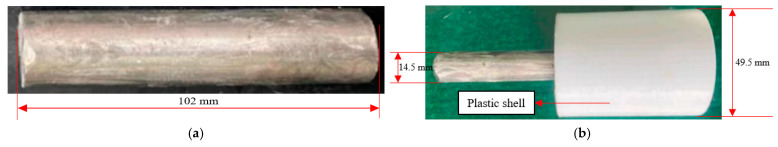
Cylindrical fragments in the tests. (**a**) Cylindrical fragment; (**b**) cylindrical fragment and plastic shell.

The shooting system used in the impact testing was a 50 mm air gun. The test specimen was mounted on a stand with a window measuring 85 × 85 mm, as illustrated in Figure 3. Impact tests with cylindrical fragments were conducted to explore the mechanism of composites against the high-velocity impact of big fragments. As illustrated in Figure 4a, the cylindrical fragments in the test measured approximately 14.5 × 102 mm and weighed approximately 130 g. To allow the cylindrical fragments to be thrown at a specific velocity, they were attached to a plastic casing. Figure 4b shows that the plastic casing measured approximately 49.5 × 60 mm. During the impact test, the cylindrical fragment had an initial velocity of about 170 m/s.

### 2.2. Test Results and Analysis

In this study, three impact tests were conducted: the target plate material for the first test was a single-layer aluminum foam material; the target plate material for the second test was an aluminum foam composite with a single-sided polyurea coating; and the target plate material for the third test was an aluminum foam composite with a double-sided polyurea coating. The single-layer aluminum foam material was penetrated in the first test, and the holes on the back side were larger than the holes on the front side. The aluminum foam composite with a single-sided polyurea coating was not penetrated in the second test, and the broken fragments of the composite during impact caused the polyurea coating on the back to bulge out in large areas. In the third test, the aluminum foam composite with a double-sided polyurea coating was penetrated, the polyurea coating on the back was fractured, and the area of the holes in the back polyurea coating was larger than the area of the holes in the front polyurea coating. The energy absorption of composite materials can be determined by the damage situation of composite materials. The specific damage of the target material in the test is shown in Table 2. The test results show that the polyurea filler material and the polyurea coating can significantly improve the impact resistance of the aluminum foam, the aluminum foam composite with back polyurea coating has the best impact resistance, the aluminum foam composite with double-sided polyurea coating has a little bit worse impact resistance than the aluminum foam composite with back polyurea coating, and the single-layer aluminum foam material has the worst impact resistance. The degree of damage to the target plate material in the three impact tests is different, and their forms of destruction are also different. The single-layer aluminum foam material in the first test was penetrated by the cylindrical fragments, and its form of damage was mainly the shear damage of the spherical cell wall. The aluminum foam composite material with back polyurea coating in the second test was mainly damaged by shear damage of spherical cell walls and polyurea filler material. The aluminum foam composite with double-sided polyurea coating in the third test was mainly damaged by shear damage of the front polyurea coating and spherical cell walls, and tensile damage of the polyurea filler material and the back polyurea coating. The damage forms of the target plate material showed that the aluminum foam/polyurea composite had better shear strength and the polyurea coating had better tensile strength. Therefore, the composite material in which the polyurea coating is a single layer and the coating is on the back has the best impact resistance in this test.

**Figure 5:**
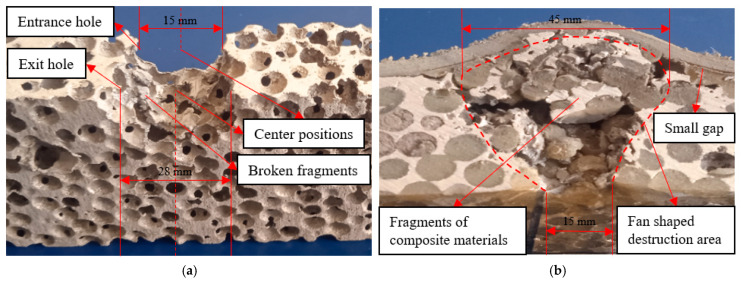
Impact test results. (**a**) Single-layer aluminum foam; (**b**) polyurea coating on the back.

As shown in Figure 5a, the single-layer aluminum foam material was severely damaged by cylindrical fragments in the impact test. The inlet and outlet diameters of the single-layer aluminum foam material were 15 mm and 28 mm, respectively. The walls of the impact holes were covered with broken fragments of the material, and most of the broken fragments were flat. During the impact process, the flight direction of the cylindrical fragments changed significantly after they passed through the single-layer aluminum foam material, and the centroid position of the back hole was shifted significantly compared with the centroid position of the front hole. The area of the back holes of the single-layer aluminum foam material is significantly larger than the area of the front holes, which is mainly due to the accumulation of spherical cell structure damage. As shown in Figure 5a, the fracture of the spherical cell wall is mainly shearing damage, in which the flattened material fragments also indicate the plastic damage of aluminum metal. Because the irregular spherical porous structure of the single-layer aluminum foam material causes discontinuous impacts of the cylindrical fragments with the spherical cells, the path of the cylindrical fragments penetrating the target plate is changed. As a result, the position of the center point of the holes on the back of the single-layer aluminum foam material was shifted compared to the position of the center point of the holes on the front, which also led to a significant change in the direction of flight of the cylindrical fragments after they penetrated the target plate. In addition, the change in the path of the cylindrical fragments through the target plate further improves the energy absorption efficiency of the single-layer aluminum foam material.

As shown in Figure 5b, the polyurea coating was located at the back of the composite, and the composite successfully resisted the impact of the cylindrical fragments. The area of the hole in the front of the composite is significantly smaller than the area of the raised area in the back, and the diameters of the hole in the front and the raised area in the back are 15 mm and 45 mm, respectively. The damage area inside the composite is roughly fan-shaped. During the impact process, the composite fragments generated by impact increased. Because the accumulation of composite fragments increased the contact area between the composite fragments and the back polyurea coating, the back polyurea coating of the composite was raised in a large area. The damaged area of the aluminum foam composite with a back polyurea coating was fan-shaped, and its main damage patterns were broken fragments of the composite and local deformation of the polyurea coating. As shown in Figure 5b, the aluminum foam at the entry hole of the composite was mainly subjected to compressive and shear stresses, while the aluminum foam in the sector-shaped damage area was mainly subjected to tensile and shear stresses. During the impact process, the broken fragments of the aluminum foam and the polyurea filler material were compressed by the cylindrical fragments and piled up on the back of the composite, increasing the contact area between the broken fragments of the composite and the polyurea coating on the back. The back polyurea coating is mainly subjected to tensile deformation because the increased contact area enables the back polyurea coating to disperse the impact stress more effectively. The back polyurea coating has good tensile properties, and the dispersion of impact stress makes the back polyurea coating less likely to be penetrated, thus improving the impact resistance of the composite. In addition, the molecular chain of the polyurea changes from the curled state to the extended state, and the extension of the molecular chain in the tensile direction leads to more impact energy dissipation. As can be seen in Figure 5b, the polyurea coating has small gaps between the polyurea coating and the skeleton in localized areas, which may lead to the deterioration of the impact resistance of the composites. Therefore, by improving the preparation process of the composites so that the bond strength between the polyurea coating and the skeleton increases, the impact resistance of the composites may be further improved.

**Figure 6:**
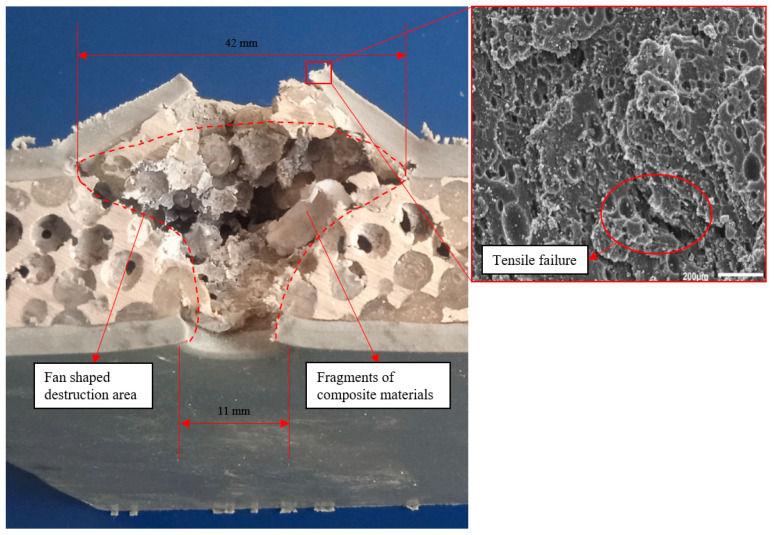
Impact test result of double-sided polyurea coating.

As shown in Figure 6, the aluminum foam composite with double-sided polyurea coating did not resist the impact of the cylindrical fragments, and some of the composite fragments flew out of the target with the cylindrical fragments during the impact. When the cylindrical fragments impacted the aluminum foam composite with double-sided polyurea coating, the front polyurea coating broke. As the number of composite fragments increased, the raised area and height of the back polyurea coating increased. When the back polyurea coating fractured, composite fragments flew out of the holes in the back polyurea coating. The damaged area inside the composite material was roughly cylindrical and fan-shaped. During the impact process, the accumulation of composite material fragments causes the raised area of the back polyurea coating to be larger than the hole area of the front polyurea coating. The diameter of the front hole is 11 mm, and the diameter of the back raised area is 42 mm. The damage region of the aluminum foam composite with double-sided polyurea coating is mainly composed of two parts: cylindrical damage region and fan-shaped damage region, and the damage forms of the front and back polyurea coatings are shear fractures and tensile fractures, respectively. As shown in Figure 6, the large compressive strength generated by the impact of the cylindrical fragments caused the front polyurea coating to shear fracture, and the polyurea coating on the front of the composite was peeled off. Because of the low shear strength of the front polyurea coating, the velocity of the cylindrical fragments was not reduced much by the blockage of the front polyurea coating. As a result, most of the spherical cells near the entry hole and the polyurea filler material undergo shear fracture, resulting in a cylindrical damage region. When the cylindrical damage region absorbed part of the impact energy, the impact velocity of the cylindrical fragments was significantly reduced. The composite material in the fan-shaped damage region mainly undergoes a tensile-shear composite deformation mode, resulting in the area of the fan-shaped damage region being significantly larger than that of the cylindrical damage region. The composite fragments produced by the destruction of the composite material are compressed by the cylindrical fragments, and the accumulation of the composite fragments increases the force area of the back polyurea coating, resulting in a larger area of holes in the back polyurea coating than in the front polyurea coating. When the back polyurea coating reached its tensile strength limit, the back polyurea coating tensile fractured. In addition, when the back polyurea coating tensile fractured, composite fragments flew out of the coating holes along with the cylindrical fragments.

## 3. Numerical Simulations

### 3.1. Geometric Models

**Figure 7:**
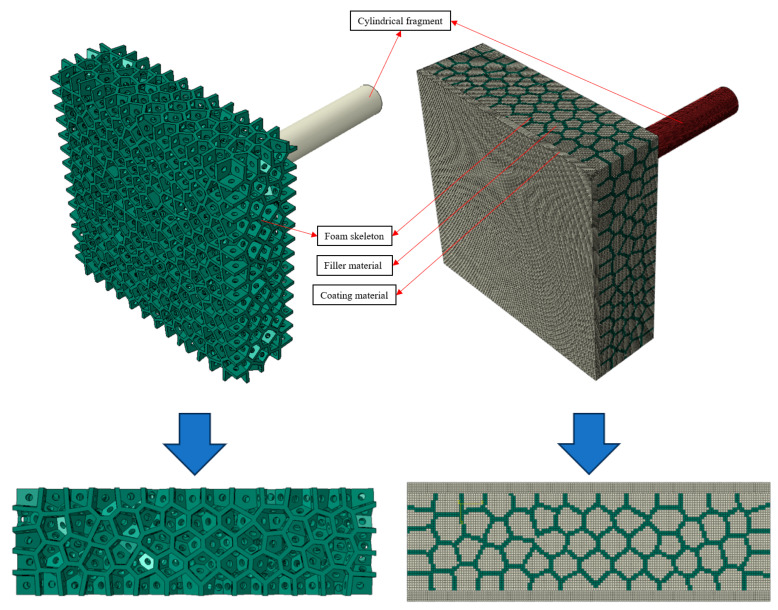
Finite element model.

To further investigate the impact resistance behavior of aluminum foam composites, numerical simulations of the impact resistance process of single-layer aluminum foam materials and aluminum foam composites were carried out. Since the single-layer aluminum foam material is an irregular spherical porous structure, the geometrical model was developed in Abaqus 2022 software through Python programming language. A closed-cell foam model similar to the actual one is established by the Voronoi algorithm [41], and then an open-cell foam model is established by opening holes on the polygonal boundary. The geometric model of the foam skeleton, shown in Figure 7, has a total size of 100 × 100 × 30 mm, a polygonal boundary width of 1.3 mm, cell diameters of about 6 mm, and open-cell diameters of 2 mm. The geometric model of the filler material is the remaining voids of the foam skeleton. The geometry of the polyurea coating was modeled as a 100 × 100 × 3 mm cuboid. The geometry of the debris was modeled as a cylinder with dimensions of 14.5 × 102 mm, which corresponded to the dimensions of the debris from the impact test. In addition, binding constraints were utilized to simulate the bonding of the polyurea coating.

### 3.2. Material Models

The effects of air, gravity, and thermal effects are not considered in the numerical simulations, considering the computational time and the actual situation. The aluminum foam composite includes two materials, pure aluminum, and polyurea, and the material of the cylindrical fragment is steel. The polyurea was modeled using the elastic-plastic model in Abaqus, which can simulate the fracture process of hard polyurea in composites. Pure aluminum and cylindrical fragments use Johnson–Cook model (J–C model for short), which can more accurately simulate the large deformation and damage process of metal materials, and it can take into account the strain rate effect of metal materials, the specific parameters of the material are shown in Table 3. Johnson–Cook failure model is part of the family of damage initiation criteria, which is the recommended technique for modeling progressive damage and failure of materials. The Johnson–Cook dynamic failure model is based on the value of the equivalent plastic strain at element integration points; failure is assumed to occur when the damage parameter exceeds 1. The damage parameter, ω, is defined as:

(1) $$\omega =\sum \frac{\Delta {\bar{\varepsilon }}^{pl}}{{\bar{\varepsilon }}_{f}^{pl}}$$

where $\Delta {\bar{\varepsilon }}^{pl}$ is an increment of the equivalent plastic strain, ${\bar{\varepsilon }}_{f}^{pl}$ is the strain at failure, and the summation is performed over all increments in the analysis. The strain at failure, ${\bar{\varepsilon }}_{f}^{pl}$, is assumed to be dependent on a nondimensional plastic strain rate, ${\frac{.}{\varepsilon }}^{pl}/{\dot{\varepsilon }}_{0}$; a dimensionless pressure-deviatoric stress ratio, *p/q* (where *p* is the pressure stress and *q* is the Mises stress); and the nondimensional temperature, $\hat{\theta }$, defined earlier in the Johnson–Cook hardening model. The dependencies are assumed to be separable and are of the form:

(2) $${\bar{\varepsilon }}_{f}^{pl}=\left[d_{1}+d_{2}\exp\left(d_{3}\frac{p}{q}\right)\right]\;\left[1+d_{4}ln\left(\frac{{\frac{.}{\varepsilon }}^{pl}}{{\dot{\varepsilon }}_{0}}\right)\right]\;\left(1+d_{5}\hat{\theta }\right)$$

where *d*_1_–*d*_5_ are failure parameters measured at or below the transition temperature, *θ*_transition_, and ${\dot{\varepsilon }}_{0}$ is the reference strain rate. This expression for ${\bar{\varepsilon }}_{f}^{pl}$ differs from the original formula published by Johnson and Cook in the sign of the parameter *d*_3_. This difference is motivated by the fact that most materials experience an increase in ${\bar{\varepsilon }}_{f}^{pl}$ with increasing pressure-deviatoric stress ratio; therefore, *d*_3_ in the above expression will usually take positive values [42].

### 3.3. Unit Algorithm

The cylindrical fragments, the aluminum foam skeleton, the polyurea filler material, and the polyurea coating are all simulated using eight-node linear hexahedral cells with high simulation accuracy because the damage processes of the cylindrical fragments and the aluminum foam composite are taken into account in the numerical simulations [45]. Since the geometric model is highly irregular, meshes need to be mapped on the model, and mesh deletion techniques are utilized to simulate the damage process. In Abaqus, mapping grid is a structured meshing technique. It is the division of a regular region of a geometric model into regular grid cells by means of specific mapping rules. The aluminum foam model is partitioned into multiple subregions in the shape of hexahedra, and the mapping mesh is applied on these subregions. In addition, the meshes of each component of the cylindrical fragments and the aluminum foam composite need to be refined as much as possible to improve the accuracy of the simulation. The size of the model is 0.65 × 0.65 × 0.65 mm, except for the polyurea-coated model, and the cell size of the polyurea-coated model is 0.5 × 0.5 × 0.5 mm. According to the conditions of the impact test, the aluminum foam composite material is fixed, and the initial velocity of the cylindrical fragment is given to 170 m/s. During the contact between the cylindrical fragments and the aluminum foam composite, both the cylindrical fragments and the aluminum foam composite are damaged. Therefore, to avoid the occurrence of mesh penetration, the specified face contact in the generic contact is defined to make the internal face contact between the cylindrical fragment and the aluminum foam composite.

### 3.4. Numerical Simulation Results

The target materials in the numerical simulation corresponded to the target materials in the test, and they included a single-layer aluminum foam material, an aluminum foam composite with a back polyurea coating, and an aluminum foam composite with a double-sided polyurea coating. The impact resistance of the target materials in the numerical simulation is shown in Table 4. The simulation results show that the polyurea filler material and the polyurea coating can effectively improve the impact resistance of the aluminum foam, and the damage of the target plate material in the simulation results is approximately the same as that of the target plate material in the test.

**Figure 8:**
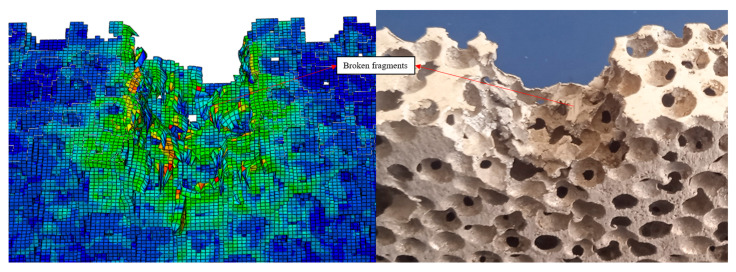
Impact simulation result of single-layer aluminum foam.

The single-layer aluminum foam material was penetrated by the cylindrical fragments, and the area of the back holes was larger than that of the front holes, and the simulation results were consistent with the test results. The velocity of the cylindrical fragments during the impact process decreased to 145 m/s at 100 μs and 129 m/s at 500 μs. The difference in the kinetic energy of the cylindrical fragments before and after the impact is 834.42 J, which is similar to the impact energy absorbed by the single-layer aluminum foam material in the previous study [46]. As shown in Figure 8, the damaged cross-section of the porous structure near the entry hole is relatively flat, the damaged cross-section of the porous structure near the exit hole is elongated, and the wall of the damaged porous cell near the exit hole is bent outward. The cylindrical fragments compressed the porous cells so that the porous cells were destroyed, and broken fragments flew out of the exit hole along with the cylindrical fragments.

**Figure 9:**
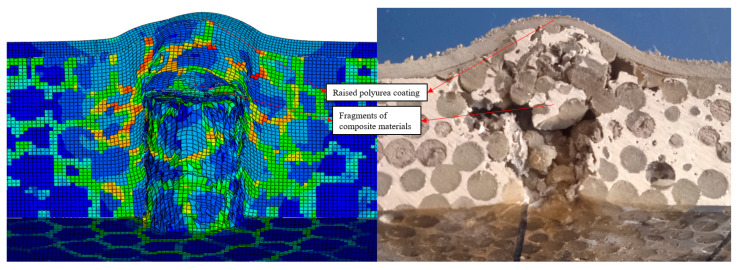
Impact simulation result of aluminum foam composite with polyurea coating on the back.

Aluminum foam composites with a back polyurea coating were not penetrated by the cylindrical fragments, and the accumulation of broken fragments raised the back polyurea coating, and the simulation results were similar to the test results. As shown in Figure 9, the porous structure and filler material fractured because of stress concentration near the entry hole. The composite fragments formed by fracture increased the area of the impact hole. The accumulated composite fragments caused the back polyurea coating to bulge and the stress concentration phenomenon occurred in the porous structure near the back polyurea coating. The velocity of the cylindrical fragments decreased to about 110 m/s at 100 μs and to 0 m/s at about 345 μs, and the cylindrical fragments after 345 μs moved in the opposite direction. In addition, the shape of the bumps in the back polyurea coating in the simulation results was similar to the shape of the bumps in the back polyurea coating in the test.

**Figure 10:**
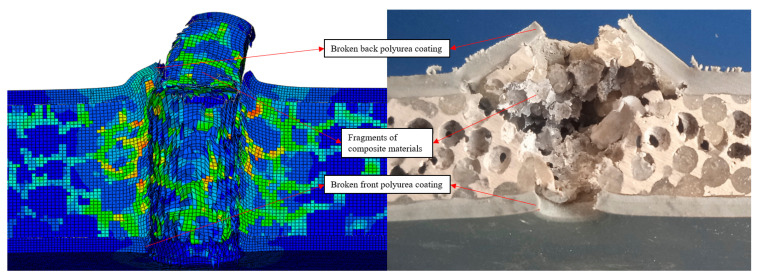
Impact simulation result of aluminum foam composite with double-sided polyurea coating.

Aluminum foam composite with double-sided polyurea coating was penetrated by cylindrical fragments, and the area of the back holes was larger than that of the front holes, and the simulation results were close to the test results. The velocity of the cylindrical fragments decreases to 112 m/s at 100 μs and t0 m/s at 500 μs. As shown in Figure 10, the front polyurea coating is fractured, and the fracture cross-section is concave. The composite fragments were compressed and piled up, and the piled-up composite fragments enlarged the area of the impact hole. The back polyurea coating was raised and then fractured, and the fracture hole was bent outward. During the impact process, stress concentration occurred in both the piled-up composite fragments and the aluminum foam near the holes.

### 3.5. Analysis of Simulation Results

**Figure 11:**
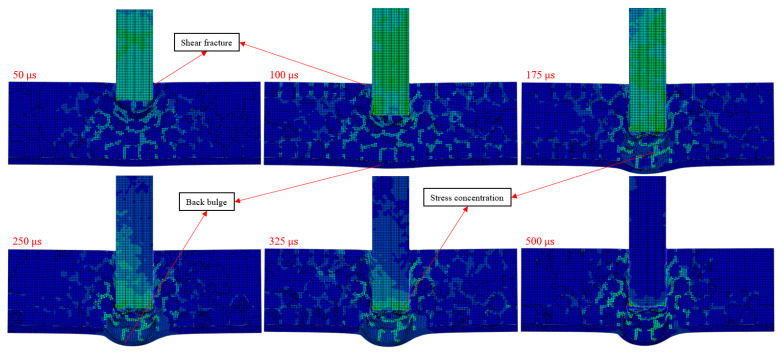
Impact resistance simulation process of aluminum foam composite with polyurea coating on the back.

Analysis of the simulation results shows that the polyurea filler material enhances the shear strength of the composite material, and the good tensile properties of the polyurea coating enable the composite material to absorb more impact energy. Because the polyurea filler material enhances the shear strength of the aluminum foam composites, the velocity difference of cylindrical fragments in the aluminum foam composites with double-sided polyurea coatings is larger than that of cylindrical fragments in the single aluminum foam material. Because the polyurea coating has good tensile properties and poor shear properties, the velocity difference of cylindrical fragments in the aluminum foam composites with back polyurea coating is larger than the velocity difference of cylindrical fragments in the aluminum foam composites with double-sided polyurea coating, the impact process is shown in Figure 11. The velocity difference of cylindrical fragments reflects the impact resistance of the composite material, and the larger the velocity difference of cylindrical fragments represents the better impact resistance of the composite material. In the impact process, the damage form of the aluminum foam composite near the entry hole is mainly shear damage. Therefore, the shear strength of the composite of filler material and aluminum foam is greater than that of the polyurea coating, and the composite filled with polyurea should be on the front and the polyurea coating should be on the back.

**Figure 12:**
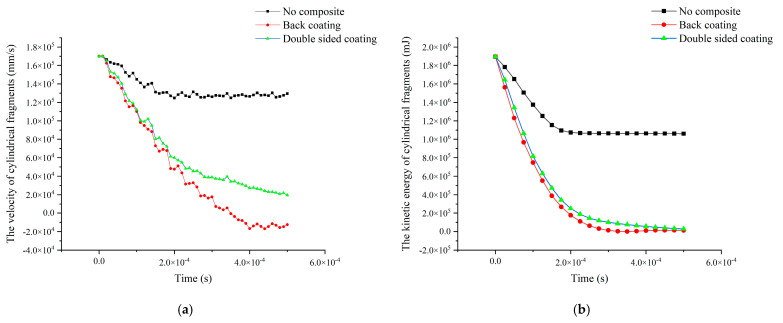
Changes in velocity and kinetic energy of cylindrical fragments with an initial velocity of 170 m/s in different target materials. (**a**) The velocity of fragments; (**b**) the kinetic energy of fragments.

The area of the holes in the back of the single-layer aluminum foam material is larger than that in the front, which is due to its better tensile resistance than shear resistance. As shown in Figure 12, the cylindrical fragments have penetrated the single-layer aluminum foam material at about 200 μs, and the cylindrical fragments stay inside the material for a short time. Therefore, the single-layer aluminum foam material absorbed very little impact energy, which was mainly due to the poor shear resistance of the single-layer aluminum foam material. Because the single-layer aluminum foam material lacked the support of the polyurea coating on the back, the single-layer aluminum foam material was easily penetrated. The single-layer aluminum foam material has long broken fragments inside, which is mainly due to the good toughness of aluminum metal. When the depth of impact increases, the stress concentration range of single-layer aluminum foam material expands. When the velocity of impact decreases, the porous structure mainly undergoes tensile damage, and the broken fragments of the material are compressed and piled up by the cylindrical fragments. As a result, the pore area inside the single-layer aluminum foam material expands during the impact process.

The aluminum foam composite with a back polyurea coating was not penetrated by the cylindrical fragments due to the reinforcement of the composite by the polyurea. There was a high impact pressure near the entry hole, which resulted in the shear fracture of the composite, and the fracture surface of the composite was flat. The impact of the cylindrical fragments produced a spherical compression wave, which resulted in stress concentration in the compressed region of the composite. The toughness of the composite material was significantly increased because of the good toughness of the polyurea filler material. As a result, the damage range of the composite material increases, and the area of the impact hole increases. The broken fragments of the composite were compressed by the cylindrical fragments, resulting in the accumulation of composite fragments on the back of the composite. Because of the buildup of the composite fragments, the back polyurea coating forms a hump-shaped bulge. The accumulation of the composite fragments increases the force area of the back polyurea coating. The back polyurea coating has good tensile properties and the increased force area makes it less likely to be penetrated. Therefore, the impact resistance of aluminum foam composites is enhanced due to the stacking of composite fragments.

Aluminum foam composites with the same thickness of double-sided polyurea coating were penetrated by the cylindrical fragments because of the low shear strength of polyurea. The large kinetic energy of the cylindrical fragments subjected the composites to a high impact pressure, causing the front polyurea coating to shear fracture. When the depth of the impact is the same, the velocity difference of the cylindrical fragments in the aluminum foam composite with a back polyurea coating is larger than that of the cylindrical fragments in the aluminum foam composite with a double-sided polyurea coating. Therefore, the shear strength of the aluminum foam filled with polyurea is greater than the shear strength of the polyurea coating, and the impact resistance of the aluminum foam composite with filler material is better than that of the polyurea coating at high impact pressures. In addition, the force area of the front polyurea coating is smaller than that of the back polyurea coating, which cannot take advantage of the fact that the polyurea coating has good toughness. When the depth of impact increases, the broken fragments of the composite material accumulate at the back polyurea coating. The accumulation of composite fragments increases the force area of the back polyurea coating and the back polyurea coating bulges. The front polyurea coating absorbed less impact energy compared to the aluminum foam/ polyurea composite. As a result, the impact pressure generated by the residual kinetic energy of the cylindrical fragments exceeded the strength limit of the back polyurea coating, and the back polyurea coating fractured. The polyurea coating on the back gives better impact resistance to the composite compared to the polyurea coating on the front of the composite.

### 3.6. Impact Resistance of Composites with Different Thickness Ratios

**Figure 13:**
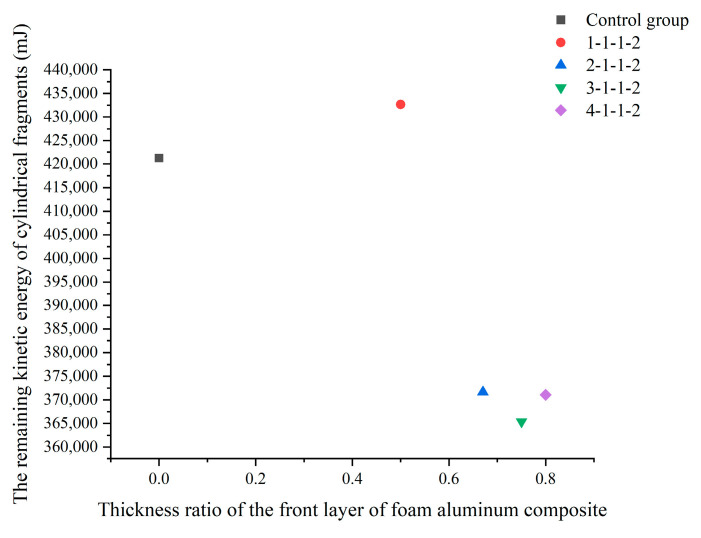
The remaining kinetic energy of cylindrical fragments in different target materials.

The accuracy of the numerical model is verified by the comparative analysis of numerical simulation results and test results. To study composites with better impact resistance, five additional simulation conditions were designed using the validated numerical model. The composite materials in four simulated working conditions were divided into four layers, two layers of aluminum foam/polyurea composite and two layers of polyurea coating. The material, porous structure, and dimensions of the composites were kept constant. The thickness ratios of the aluminum foam filled with polyurea composite are 1:1, 2:1, 3:1, and 4:1, respectively, and the ratio of the thickness of the polyurea layer in the middle of the composite to the thickness of the polyurea coating on the back is 1:2 (the composites with different thickness ratios are represented by the form of 1-1-1-2, with the first two digits representing the ratio of the thickness of the aluminum foam filled with polyurea composite and the last two digits representing the ratio of the thickness of the polyurea coating). To investigate whether the new structure is effective in enhancing the impact resistance of the composites, the aluminum foam composites with back polyurea coating were used as the control group. The impact velocity was 200 m/s in the numerical simulation to compare the impact resistance and energy absorption efficiency of composites with different thickness ratios. During the impact process, the velocity loss of the cylindrical fragments is not sufficient to account for the energy consumption of the cylindrical fragments because the mass loss of the cylindrical fragments is different in different thickness ratios of composites. Therefore, the residual kinetic energy of the cylindrical fragments was used as a measure of the energy absorption efficiency of the composites, and the velocity difference of the cylindrical fragments was used as a measure of the impact resistance of the composites. The remaining kinetic energy of the cylindrical fragments is shown in Figure 13.

**Figure 14:**
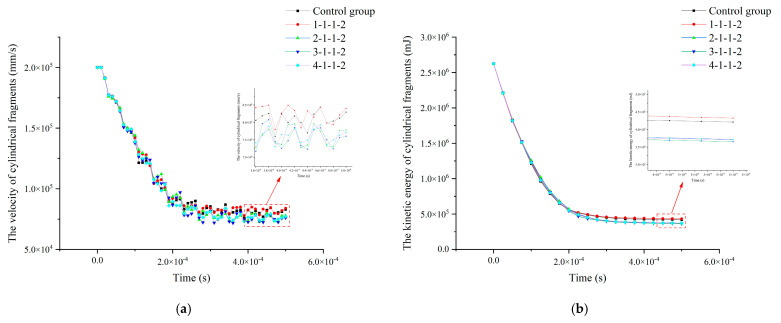
Changes in velocity and kinetic energy of cylindrical fragments with an initial velocity of 200m/s in different target materials. (**a**) The velocity of fragments; (**b**) the kinetic energy of fragments.

The velocity profiles of the cylindrical fragments are shown in Figure 14a, and the residual velocity of the cylindrical fragments is minimized in the type 3-1-1-2 composite. The residual velocities of the cylindrical fragments in the 4-1-1-2 composites were smaller than those in the 2-1-1-2 composites, and the residual velocities of the cylindrical fragments in the 1-1-1-2 composites were the largest and larger than those of the cylindrical fragments in the controls. The kinetic energy curves of the cylindrical fragments are shown in Figure 14b, and the decreasing trend of the kinetic energy of the cylindrical fragments in composites with different thickness ratios is the same. The residual kinetic energy of the cylindrical fragments in the 3-1-1-2 composites was the smallest, the residual kinetic energy of the cylindrical fragments in the 4-1-1-2 composites was smaller than that of the cylindrical fragments in the 2-1-1-2 composites, and the residual kinetic energy of the cylindrical fragments in the 1-1-1-2 composites was the largest and larger than that of the cylindrical fragments in the control group. The impact resistance of different aluminum foam composites is shown in Table 5. Compared to the residual kinetic energy of the cylindrical fragments in the control group, the residual kinetic energy of the cylindrical fragments in the 3-1-1-2 composite decreased by 13.26%, the residual kinetic energy of the cylindrical fragments in the 4-1-1-2 composite decreased by 11.91%, and the residual kinetic energy of the cylindrical fragments in the 2-1-1-2 composite decreased by 11.78%. However, the residual kinetic energy of the cylindrical fragments in the 1-1-1-2 composite was increased by 2.7% compared to the control group.

**Figure 15:**
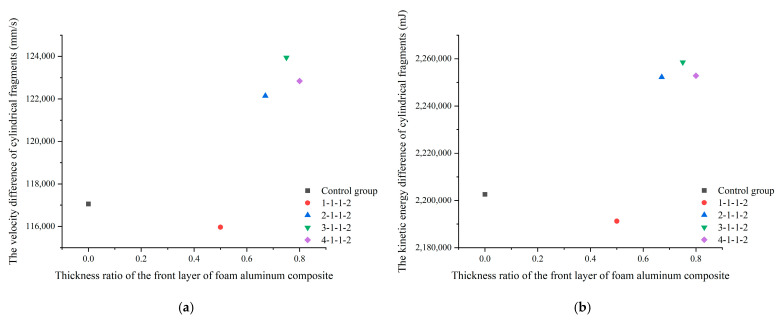
The velocity and kinetic energy differences of cylindrical fragments in different target materials. (**a**) The difference of velocity; (**b**) the difference of kinetic energy.

Analysis of the simulation results shows that the thickness ratio of the aluminum foam/polyurea composite is not linearly related to the impact resistance of the composite, and the thickness ratio of the aluminum foam/polyurea composite has an optimal value. The velocity difference of the cylindrical fragments is shown in Figure 15a, and when the thickness of the front layer of the aluminum foam/polyurea composite increases, the velocity difference of the fragments first increases and then decreases. The kinetic energy difference of the cylindrical fragments is shown in Figure 15b, which increases and then decreases when the thickness of the front layer of the aluminum foam/polyurea composite increases. Therefore, the combination of the scatter plot of the kinetic energy difference and the scatter plot of the velocity difference shows that the 3-1-1-2-type composite has the best impact resistance and the highest energy absorption efficiency. In the practical preparation of aluminum foam composites for impact resistance, the application of aluminum foam composites of type 3-1-1-2 can be considered.

## 4. Conclusions

In this paper, the impact resistance of aluminum foam composites containing fillers and coatings is investigated experimentally and through numerical simulations. The impact resistance and energy absorption of aluminum foam composites with different thickness ratios are analyzed using a validated numerical model, and the following conclusions are obtained:

(1) Polyurea filler material and polyurea coating can effectively improve the impact resistance of aluminum foam, and the composite with coating on the back has the best impact resistance. The polyurea filler material resulted in enhanced shear strength of the aluminum foam composites. The aluminum foam composites with greater shear strength had a greater range of internal damage compared to the single-layer aluminum foam material without polyurea filler, allowing the composites to absorb more impact energy. The polyurea coating has good tensile properties and has a good supporting role in the aluminum foam composite. The buildup of the aluminum foam composite fragments increases the force area of the back polyurea coating, resulting in the polyurea coating dispersing more of the impact stress, which improves the impact resistance of the aluminum foam composite. Therefore, the impact resistance of aluminum foam composites with double-sided polyurea coatings is somewhat worse than that of aluminum foam composites with back polyurea coatings, the single-layer aluminum foam material had the worst impact resistance.
(2) For the energy absorption effect of aluminum foam composites containing fillers and coatings during impact resistance, there is an optimal value for the thickness ratio of aluminum foam/polyurea composite, which is 3:1. The Abaqus numerical model verified by the test results was used to simulate the aluminum foam composites with different thickness ratios and the control group. Compared to the residual kinetic energy of the cylindrical fragments in the control group, the residual kinetic energy of the cylindrical fragments in the 3-1-1-2 composite decreased by 13.26%, the residual kinetic energy of the cylindrical fragments in the 4-1-1-2 composite decreased by 11.91%, and the residual kinetic energy of the cylindrical fragments in the 2-1-1-2 composite decreased by 11.78%. However, the residual kinetic energy of the cylindrical fragments in the 1-1-1-2 composite was increased by 2.7% compared to the control group. From the scatter plots of the kinetic energy difference and velocity difference of the cylindrical fragments, it can be seen that, when the thickness of the previous layer of the aluminum foam/ polyurea composite increases, the kinetic energy difference and velocity difference of the cylindrical fragments both increase first and then decrease. Combined with the scatter plot of the residual kinetic energy and the scatter plot of the velocity difference of the cylindrical fragments, it can be seen that the type 3-1-1-2 composite material has the best impact resistance and the highest efficiency of absorbing energy. The type 3-1-1-2 composite material can be used as a reference for the design of impact-resistant composite materials in the future.

## Figures and Tables

**Table 1:** Parameters of three types of polyurea.

Polyurea	Density/ (g/cm^3^)	Tensile Strength/ (MPa)	Tear Strength/ (kN/m)	Elongation
APC-40	1.07	32	110	≥300

**Table 2:** Data of test results.

Test Number	Sample Number	Materials	The Diameter of the Front Hole/(mm)	Damage Area of the Front Surface/(mm^2^)	Damage Area of the Back Surface/(mm^2^)
Test 1#	1	No composite	15	176.63	615.44
Test 2#	1	Back coating	15	176.63	1589.63
Test 3#	1	Double-sided coating	11	94.99	1384.74

**Table 3:** Material parameters of cylindrical fragments and aluminum matrix.

Constants	Steel [43]	Aluminum [44]
Density, (kg/m^3^)	7800	2712.6
Modulus of Elasticity, E (GPa)	200	68.948
Static yield strength, A (GPa)	0.507	0.10282
Strain hardening coefficient, B(GPa)	0.32	0.04979
Strain hardening exponent, n	0.28	0.197
Strain rate coefficient, C	0.064	0.001
Reference Strain rate, $\varepsilon_{0}$(s^−1^)	1	1
Thermal softening exponent, m	1.06	0.859
Reference temperature, t0 (K)	298	293
Melting temperature, tm (K)	1795	893
Damage constant, D_l_	0.1	0.071
Damage constant, D_2_	0.76	1.248
Damage constant, D_3_	1.57	−1.142
Damage constant, D_4_	0.005	0.147
Damage constant, D_5_	−0.84	0

**Table 4:** Numerical simulation results.

Test Materials	Initial Speed/ (mm/s)	Residual Velocity/ (mm/s)	Speed Difference/ (mm/s)	Absorbed Energy/ mJ
No composite	170,000	129,499	40,551	834,420
Back coating	170,000	0	170,000	1,895,800
Double-sided coating	170,000	19,520	150,480	1,866,562

**Table 5:** Numerical simulation results of extended research.

Conditions	Simulation Materials	Initial Speed/ (mm/s)	Initial Kinetic Energy/ mJ	Speed Difference/ (mm/s)	Kinetic Energy Difference/ mJ	Remaining Kinetic Energy/ mJ
Case 1#	Control group	200,000	2,623,870	117,053	2,202,612	421,258
Case 2#	1-1-1-2	200,000	2,623,870	115,965	2,191,222	432,648
Case 3#	2-1-1-2	200,000	2,623,870	122,150	2,252,216	371,654
Case 4#	3-1-1-2	200,000	2,623,870	123,942	2,258,489	365,381
Case 5#	4-1-1-2	200,000	2,623,870	122,844	2,252,829	371,061

## Data Availability

Data are contained within the article.
